# Book Reviews

**Published:** 1902-10

**Authors:** 


The Operations of Surgery. By W. H. A. Jacobson, M. Ch., Oxon, F. R. C. S., Surgeon to Guy's Hospital; Consulting Surgeon, Royal Hospital for Children and Women; Member Court of Examiners, Royal College of Surgeons, etc; and F. J. Stewart, M. S., London, F. R. C. S., Assistant Surgeon, Guy's Hospital and to the Hospital for Sick Children; Surgeon-in-Charge of the Throat Department, Guy's Hospital. Fourth edition, revised, enlarged, and improved. Five hundred and fifty illustrations. Two volumes. Volume I: Operations on the Upper Extremity, Operations on the Head and Neck, Operations on the Thorax. Volume II: Operations on the Abdomen, Operations on the Lower Extremity, Operations on the Vertebral Column. Philadelphia: P. Blakiston's Son & Co. 1902. [Price, cloth, $10.00; sheep, $12.00 net.]

This author is no stranger to the medical profession of this country. He has long been recognised as one of the leading surgeons of Great Britain and his "Operations of Surgery" took appropriate place when the book first appeared, now some fifteen years ago, among the best modern surgical treatises. Each succeeding edition has only further accentuated the fact that the author is a thoroughly conscientious exponent of the most modern principles of surgical art and science. He writes clearly, though concisely, and uses scarcely a superfluous word, thereby making his treatise the more readable and the easier to be remembered.

Beginning with the preface we desire to invite attention to it as a model. The author tells first when each of the three preceding editions of his book appeared—an item of time-saving assistance. We have spent valuable time on many occasions in search of the information contained in the first two lines of this preface, a piece of intelligence that most authors of late seem inclined to hide. Moreover, the entire preface conveys the precise information that every one interested desires, and especially a reviewer.

Mr. Jacobson has been slow to put forth this edition for the reason, among others, that he finds it increasingly difficult as time goes on, to make it express the surgical conditions of today, each result being disappointing in that regard. No doubt this is the experience of most authors with new editions, but especially so in the surgical field. Nevertheless, he has given us a surprisingly up-to-date work, and has spared no pains in time or labor, has omitted no reference or illustration that would add to the usefulness of his book. The increase in the number of engravings has been very large and the author has given this part of the work personal attention. Its importance cannot be overestimated; good illustrations make the text clearer, poor ones only obscure it.

It should be borne in mind that this work is intended particularly "for the use of those recently appointed on a hospital staff, and for those preparing for the higher examinations." These are the author's own words and form a part of the title page, and Jacobson emphasises this point with some fervor in his preface. We take pleasure in adding that it is a splendid work for the general practitioner of medicine residing where he must needs do much surgery. Furthermore, it is a treatise that no teacher of surgery and no one who practises surgery exclusively can afford to neglect or go without. In two volumes it is a handsome, useful, and necessary addition to the surgeon's library.

A PRACTICAL MANUAL OF BACTERIOLOGY FOR STUDENTS AND PHYSICIANS By A. C. ABBOTT, M. D., Professor of Hygiene, University of Pennsylvania. New (6th) edition, revised and enlarged. In one 12mo volume of 636 pages, with 111 illustrations, 26 colored. Philadelphia and New York: Lea Brothers & Co. 1902. [Price, cloth, $2.75 net.]

The remarkable growth in bacteriological research, especially within the last few years, has rendered it necessary to recast much of the literature on the subject. The popularity of Abbott's work began with its first issue in 1891 and has steadily increased during the five editions that appeared within eight years. It is now three years since the fifth edition was sent out and during that time bacteriology has advanced materially in several directions. Some of the important of these are that new light has been shed upon the etiology of epidemic cerebro-spinal meningitis and dysentery. The study of tuberculosis, too, has become of vast interest, leading to the discovery of a new group of microörganisms closely allied to the bacillus tuberculosis and apparently having the property of causing conditions quite suggestive of tubercular disease. In short, there is much advance in our knowledge of the mechanism of infection and immunity.

In this edition Abbott has brought forward the study to embrace the immediate present. The book has become standard as a working guide with student and teacher and is yet without a rival in the field it essays to occupy.

---

THE PRACTICAL MEDICINE SERIES OF YEAR BOOKS. Ten volumes. Issued monthly under the general editorial charge of Gustavus P. Head, M. D., Professor of Laryngology and Rhinology in the Chicago Post-Graduate Medical School. Volume V. Obstetrics. Edited by REUBEN PETERSON, A. B., M. D., Professor of Obstetrics and Gynecology in the University of Michigan; and HENRY F. LEWIS, A. B., M. D., Instructor in Obstetrics and Gynecology in Rush Medical College. Duodecimo, pp. 223. Illustrated. Chicago: The Year Book Publishers. 1902. [Price of this volume, $1.25; entire set, $7.50.]

This book is one of the most systematic, as well as one of the best of the series thus far issued. It sets forth the principal writings of the year pertaining to obstetrics. The topics have been judiciously arranged and the selections have been well made.

Considerable literature on obstetrical subjects has been published of late showing a swaying of the pendulum of medical science on and toward that important branch. Probably as much advancement has taken place within the last lustrum or two in the department of obstetrics as in any branch of medicine, if we judge by the life-saving results. Nowadays we do not hear of "child-bed fever," and seldom even of puerperal eclampsia, while death from postpartum hemorrhage is almost unknown; and these constitute the principal sources of danger during the puerperium.

There is considerable information to be derived in this book on all the essentials relating to the care of the puerperal woman, and the management of labor. It is not only one of the most important books of the practical medicine series, as we stated at the outset, but it is decidedly the best small work of the year for the general practitioner of medicine.

---

THE PRACTICAL MEDICINE SERIES OF YEAR BOOKS. Ten volumes. Issued monthly under the general editorial charge of Gustavus P. Head, M. D., Professor of Larngology and Rhinology in the Chicago Post-Graduate Medical School. Volume VI. General Medicine. Edited by FRANK BILLINGS, M. S., M. D., Head of Medical Department and Dean of the Faculty of Rush Medical College, Chicago. With the Collaboration of S. C. Stanton, M. D. 12mo, pp. 271. Chicago: The Year Book Publishers. 1902. [Price, $1.50.; entire series, $7.50.]

This volume deals with subjects of interest to every practitioner of internal medicine. Beginning with typhoid fever it takes up in regular order of sequence malaria and other tropical fevers and endemic diseases; the diseases of the liver, of the pancreas, of the esophagus, of the stomach, of the intestines, and, finally, parasitic diseases. The abstracts cover a vast amount of literature, they are very full and do not, as is too often the case, abridge the views of the authors who are quoted. The journals of late have been replete with gastrointestinal material and all of value has been utilised in this book. It is a credit to the distinguished editor of this volume, as well as to the editor-in-chief of the series, and will be accepted by all who are interested in its subject matter, as one of the best year-books in general medicine that has recently appeared.

---

TRANSACTIONS OF THE SOUTHERN SURGICAL AND GYNECOLOGICAL ASSOCIATION. Fourteenth Annual Meeting held at Richmond, Va., November 12, 13 and 14, 1901. W. D. Haggard, M. D., Secretary. Published by the Association. 1902.

We have had frequent occasion to mention the excellent work of this model society, as exemplified by the volume of transactions which it puts forth year by year. The present book is no exception to the custom of the association; indeed, in some respects, it may take rank as at least one of its very best publications. Primarily, the place of meeting—Richmond—contributed in a very large degree to the interest in, success of, and large attendance upon this annual session. No individual, no society ever goes to Richmond, but desires to go again. The people of that historic city receive its guests, as the Hon. Mr. Hunton said in his address of welcome, with open arms whether they come from east or west, north or south. Not only this, but their hearts are large and their latch-strings work easily.

This book, from title to index, is full of interest and is a credit to the society and especially to its accomplished secretary. The meeting this autumn will be held at Cincinnati, under the presidency of Dr. W. E. B. Davis, who organised the association, and to whose energy and executive ability are due its present strength and efficiency.

---

TWENTY-FIRST ANNUAL REPORT OF THE STATE BOARD OF HEALTH OF NEW YORK. For the year ending December 31, 1900. Transmitted to the legislature February 18, 1901. With maps. Albany: James B. Lyon, State Printer. 1901.

Upon attaining its majority the state board of health disappears and is replaced by the state commissioner of health. This, therefore, will be the last report of the board as such, but in the succeeding years, the commissioner will present the annual report of the department of health. The president of the retiring board became the commissioner, so the previous efficiency of the department will be maintained under the administration of Dr. Daniel Lewis.

This report follows the line of preceding years in presenting first a summary of the work done, signed by the board; next the sanitary status of the several important localities, pointing out epidemic diseases and the action of the board to abate them; and, finally, an appendix volume containing maps of villages and towns where public improvements, sewers, etc., have been authorised. It is a report of interest to the localities affected by the board's action, as well as to sanitarians in general.

---

THE MEDICAL TREATMENT OF GALLSTONES. By J. H. KEAY, M. A., M. D. Duodecimo, pp. 133. Philadelphia: P. Blakiston's Son & Co. 1902. [Price, $1.25]

It is coming to be the general belief among well-informed medical men that a person suffering from gallstones at once falls under the domain of surgery. In this belief we most heartily concur, notwithstanding very many strong statements in this little brochure that tend to support the medical treatment of gallstone disease. The author presents the subject in a very fair light, and we commend the book to everyone interested in the study of biliary calculi and the disturbances they engender.

